# Soluble Receptor for Advanced Glycation End Product Ameliorates Chronic Intermittent Hypoxia Induced Renal Injury, Inflammation, and Apoptosis via P38/JNK Signaling Pathways

**DOI:** 10.1155/2016/1015390

**Published:** 2016-09-05

**Authors:** Xu Wu, Wenyu Gu, Huan Lu, Chengying Liu, Biyun Yu, Hui Xu, Yaodong Tang, Shanqun Li, Jian Zhou, Chuan Shao

**Affiliations:** ^1^Department of Pulmonary Medicine, Zhongshan Hospital, Fudan University, Shanghai 200032, China; ^2^Clinical Center for Sleep Breathing Disorder and Snoring, Zhongshan Hospital, Fudan University, Shanghai 200032, China; ^3^Department of Urology, Shanghai Tenth People's Hospital, Tongji University School of Medicine, Shanghai 200072, China; ^4^Department of Respiratory Medicine, Affiliated Jiangyin Hospital of Southeast University, Jiangyin 214400, China; ^5^Department of Respiratory Medicine, Ningbo Medical Center Lihuili Eastern Hospital, Taipei Medical University Ningbo Medical Center, Ningbo 315040, China

## Abstract

Obstructive sleep apnea (OSA) associated chronic kidney disease is mainly caused by chronic intermittent hypoxia (CIH) triggered tissue damage. Receptor for advanced glycation end product (RAGE) and its ligand high mobility group box 1 (HMGB1) are expressed on renal cells and mediate inflammatory responses in OSA-related diseases. To determine their roles in CIH-induced renal injury, soluble RAGE (sRAGE), the RAGE neutralizing antibody, was intravenously administered in a CIH model. We also evaluated the effect of sRAGE on inflammation and apoptosis. Rats were divided into four groups: (1) normal air (NA), (2) CIH, (3) CIH+sRAGE, and (4) NA+sRAGE. Our results showed that CIH accelerated renal histological injury and upregulated RAGE-HMGB1 levels involving inflammatory (NF-*κ*B, TNF-*α*, and IL-6), apoptotic (Bcl-2/Bax), and mitogen-activated protein kinases (phosphorylation of P38, ERK, and JNK) signal transduction pathways, which were abolished by sRAGE but p-ERK. Furthermore, sRAGE ameliorated renal dysfunction by attenuating tubular endothelial apoptosis determined by immunofluorescence staining of CD31 and TUNEL. These findings suggested that RAGE-HMGB1 activated chronic inflammatory transduction cascades that contributed to the pathogenesis of the CIH-induced renal injury. Inhibition of RAGE ligand interaction by sRAGE provided a therapeutic potential for CIH-induced renal injury, inflammation, and apoptosis through P38 and JNK pathways.

## 1. Introduction

Obstructive sleep apnea (OSA) is characterized by repetitive upper airway collapse and recurrent hypoxia during sleep. Emerging evidence indicates that chronic kidney disease (CKD) is highly prevalent complication of untreated OSA with symptoms of polyuria and proteinuria [1, 2]. Meanwhile, the prevalence of OSA in CKD patients ranges severalfold higher than the general population [3]. Two mechanisms are responsible for the loss of kidney function in OSA patients: chronic nocturnal intrarenal hypoxia and activation of sympathetic nervous system in response to oxidative stress, resulting in tubulointerstitial injury and ultimately leading common pathway to end-stage renal disease (ESKD) [4, 5]. As the foremost pathophysiological change in the process of OSA, chronic intermittent hypoxia (CIH) often causes oxidative stress and inflammations, contributing to damage of various tissue and organs [6].

The receptor for advanced glycation end products (RAGE), first identified as a member of the immunoglobulin superfamily, is a pattern-recognition receptor that interacts with multiligands, such as advanced glycation end products (AGEs), high mobility group box 1 (HMGB1), S-100 calcium-binding protein (S100B), and Mac-1 [7]. Multiple descriptive studies have demonstrated RAGE and its ligands are potentially related to OSA. Regarding RAGE ligands, a previous study had evaluated levels of HMGB1 and their relation to endothelial function in OSA patients [8]. S100B levels, identified as a useful biochemical marker, have also been found increased in OSA [9]. Broadly speaking, RAGE and its ligands are almost expressed in all tissues and on a wide range of cell types, also including renal (proximal) tubules, mesangial cells, and podocytes [10]. More recently, accumulations of RAGE and its ligands are recognized to be upregulated in various types of renal disorders. A review investigated that RAGE was associated not only with diabetic nephropathy, but also with obesity-related glomerulopathy, hypertensive nephropathy, and ischemic renal injury, all of which were closely related to OSA-associated-renal injury [11]. Considering chronic kidney disease is an immune inflammatory condition [12], it is natural to link chronic kidney disease to RAGE-HMGB1 and to identify them as key mediators in inflammatory responses as well as potential signaling molecules in progression to ESKD [13, 14]. As expected, HMGB1 is elevated significantly in CKD patients and correlates with GFR as well as markers of inflammation [15]. In particular, serum levels of HMGB1 in CKD patients were also significantly higher than those in control subjects [16]. These findings raise the possibility of RAGE-HMGB1 in the pathogenesis of OSA-associated chronic kidney disease, but their contribution in CIH-induced renal injury has not yet been elucidated.

Furthermore, it is well documented that RAGE is an inverse marker in CKD patients [17], thus inhibition of RAGE constituting a possible strategy for the treatment of CKD [18, 19]. Soluble RAGE (sRAGE), which possesses the RAGE ligand binding positions but lacks the cytoplasmic and transmembrane domains, secretes out of the cells and acts as decoys to prevent RAGE signal transduction directly [20]. In clinical settings, serum sRAGE showed increased levels in patients with ESKD [21], but whether it could protect against toxic effects of RAGE remains to be known. Recent study found that RAGE and nuclear factor kappa B (NF-*κ*B) downstream signaling were centrally involved in sleep apnea obtained from the intermittent hypoxia (IH) experimental model [22]. In this study we establish a CIH model to investigate the participation of RAGE-HMGB1 and the therapeutic effect of recombinant soluble RAGE. The possible mechanism involved was also elucidated.

## 2. Material and Methods

### 2.1. Animal Model of CIH

 Male Sprague-Dawley rats at age of 4 weeks and body weight 140–150 g, obtained from the Experimental Animal Centre of Fudan University (China) and allowed free access to laboratory chow and tap water in day-night quarters at 25°C, were used in this study. The animal protocol was approved by the Animal Care Committee of Fudan University, in accordance with the National Institutes of Health Guide for the Care and Use of Laboratory Animals. All effects were made to minimize animal suffering. Rats were randomly divided into the following four experimental groups of 6 animals each: the normal air (NA) control group; the CIH group; the CIH plus sRAGE group; normal air plus sRAGE group (NA+sRAGE). The CIH protocol was modeled according to the study of Fu et al. [23]. Rats were placed in four identical designed chambers. Nitrogen (100%) was delivered to the chambers for 30 s to reduce the ambient fraction of inspired oxygen to 6-7% for 10 s. Then, oxygen was infused for 20 s so that the oxygen concentration returned to 20~21%. This cycle took one minute, 8 h/day for 7 d/week for 5 weeks. The oxygen concentration was measured automatically using an oxygen analyzer (Chang Ai Electronic Science & Technology Company, Shanghai, China). The CIH plus sRAGE treatment group received the same CIH protocol as for comparison with the CIH model group. Rats in the sRAGE treatment group were subsequently injected with recombinant sRAGE protein 150 *μ*g/per rat (diluted in 1 mL phosphate-buffered saline) intraperitoneally every 48 h for 5 weeks before each hypoxia cycle. This dose of rats was converted based on previous work where daily dose of sRAGE in a mouse model of chronic hypoxia was 20 *μ*g/day [24]. Control rats included NA and NA+sRAGE group rats, all of which were subjected to normal air and administered with phosphate-buffered saline (PBS) as a vehicle control and 150 *μ*g/per rat of recombinant sRAGE, respectively. During this exposure, all rats were kept under pathogen-free conditions and allowed to access to food and water. At the end of 5 weeks of CIH model, all the rats were euthanized 15 h after last hypoxia circle. Their blood and kidneys were collected. Blood samples were obtained from the inferior vena cava. Renal function was calculated as serum creatinine and blood urea nitrogen (BUN), which were measured in the core laboratory of Zhongshan Hospital (Shanghai, China).

### 2.2. Kidney Histology

After being euthanized, kidneys were fixed with 4% paraformaldehyde and embedded in paraffin. Paraffin-embedded specimens were cut into 4 *μ*m thick sections and stained with hematoxylin-eosin. Kidney injury was examined using the modified 0–5 Jablonski grading scale under a light microscope: 0 represents normal; 1 represents occasional degeneration and necrosis of individual cells; 2 represents degenerative cells and necrosis of individual tubules; 3 represents degeneration and necrosis of all cells in adjacent proximal convoluted tubules with survival of surrounding tubules; 4 represents necrosis confined to the distal third of the proximal convoluted tubules, with a band of necrosis extending across the inner cortex; and 5 represents necrosis affecting all the three segments of the proximal convoluted tubules, as described previously [25].

### 2.3. HMGB1 Immunohistochemistry

For HMGB1 detection, the samples were dewaxed in xylene and dehydrated in a graded ethanol series. Endogenous peroxidase activity was inhibited by incubating the slides with 0.3% H_2_O_2_ for 5 min, followed by washing thrice with PBS; the sections were incubated with the primary antibodies to HMGB1 (1 : 1000 dilution, ab18256; Abcam, Cambridge, UK) and incubated at 4°C overnight, washed in PBS, and incubated at 37°C for 1 h with biotinylated anti-rabbit/rat IgG (1 : 200; Maixin-Bio, Shanghai, China) according to manufacturer's instructions. The tissue was incubated with Streptavidin Peroxidase (Maixin-Bio) reagents at 37°C for 30 min, stained with freshly prepared DAB (Maixin-Bio). Morphometric quantification of the stained sections was performed with a customized digital image analysis system (IMAGE-Pro plus 4.5). Analysis of the kidney and capturing images was performed.

### 2.4. Immunofluorescent Triple Staining for CD31, TUNEL, DAPI, and Apoptotic Determination

To visualize apoptotic changes during CIH-induced renal injury, 4 *μ*m paraffin-embedded tissue slides were deparaffinized, rehydrated, and prepared as described in immunohistochemistry. Antigen was retrieved by microwave-citrate buffer antigen retrieval method. The slides were blocked with 5% goat serum (Invitrogen) for 1 hour at room temperature, permeabilized with 0.2% Triton X-100, incubated overnight at 4°C with mouse anti-rat CD31 antibody (1 : 50 dilution, No. ab64543; Abcam, Cambridge, UK). After the samples were rinsed 4 times (3 min each) with PBS, the slides were then incubated for 30 minutes at room temperature with Alexa Fluor 488-conjugated goat anti-mouse IgG (1 : 400; B40941, Invitrogen). For detection of apoptotic cells, TUNEL staining was carried out using a Promega apoptosis detection kit. Immunofluorescence for TUNEL staining was performed with Alexa Fluor 594-conjugated goat anti-mouse IgG (1 : 400; A11020, Invitrogen). The glass was mounted with cover slips containing Vectashield mounting medium with 4′,6-diamidino-2-phenylindole (DAPI; Vector Laboratories) and imaged under an fluorescent microscope (Leica). Endothelial cells (CD31+) stained green fluorescence and DAPI stained blue fluorescence. TUNEL-positive apoptotic cells were detected by localized red fluorescence within cell nuclei. TUNEL-positive (TUNEL^+^) and DAPI positive (DAPI^+^) cells were counted at ×100 magnification with a fluorescence microscopy, respectively. The number of apoptotic cells was calculated as TUNEL^+^/DAPI^+^ cells in random 10 fields per section for quantification.

### 2.5. Western Blot Analysis for Target Protein

Proteins were extracted from animal kidney tissues using NucleoSpin (REF 740933.50; Macherey-Nagel), after which they were separated with SDS-PAGE on 8% gels and transferred to PVDF membranes which were then incubated overnight at 4°C with the primary antibody diluted in blocking solution. The primary antibodies and the dilutions were as follows: p-ERK [1/2] No. 9101 (1 : 1000), p-JNK No. 9255 (1 : 2000), t-JNK No. 9252 (1 : 1000), p-p38 No. 9211 (1 : 1000), and t-p38 No. 9212 (1 : 1000) (Cell Signaling Technology, Danvers, MA); Bax No. ab5714 (1 : 500), Bcl-2 No. ab136285 (1 : 500), NF-*κ*B p65 No. ab16502 (1 : 1000), HMGB1 No. ab18256 (1 : 1000), (Abcam, Cambridge, UK), and RAGE (1 : 500, No. R3611, Sigma, USA). Horseradish peroxidase-coupled rabbit and mouse IgG (1 : 2000) were used as secondary antibodies. The blots were incubated with horseradish peroxidase-conjugated anti-IgG for 1 h at 37°C. Nonspecific binding sites were blocked for 1 h with 0.05 g/mL nonfat milk powder in Tris-buffered saline (pH 7.4) and 0.05% (v/v) Tween 20 (Bio-Rad) followed by overnight incubations with primary antibody. Blots were probed with anti-glyceraldehyde 3-phosphate dehydrogenase (GAPDH) antibody (sc-25778, Santa Cruz, USA) to ensure equal loading and detected using ECL chemiluminescent system (Amersham Biosciences, Piscataway, NJ, USA). Band intensity was quantified by scanning densitometry. Each measurement was made 3 times.

### 2.6. Enzyme-Linked Immunosorbent Assay (ELISA)

Serum was isolated from the blood after centrifugation at 14 000 rpm for 20 min at 4°C. After centrifugation, serum was frozen at −80°C until enzyme-linked immunosorbent assay (ELISA) analyses were performed. HMGB1 (IBL International) and the levels of inflammatory mediators (TNF-a, IL-6, and IL-17 from R&D Systems) in the serum samples were measured in triplicate following the procedures supplied by the manufacturer.

### 2.7. Statistical Analysis

Data were presented as mean ± SEM and analyzed using SPSS 18.0. Comparisons between multiple groups were performed using ANOVA with the Bonferroni test. *P* < 0.05 was considered statistically significant between groups.

## 3. Results

### 3.1. Protective Effect of sRAGE on Kidney Function and Histopathological Assessment

**Figure 1:**
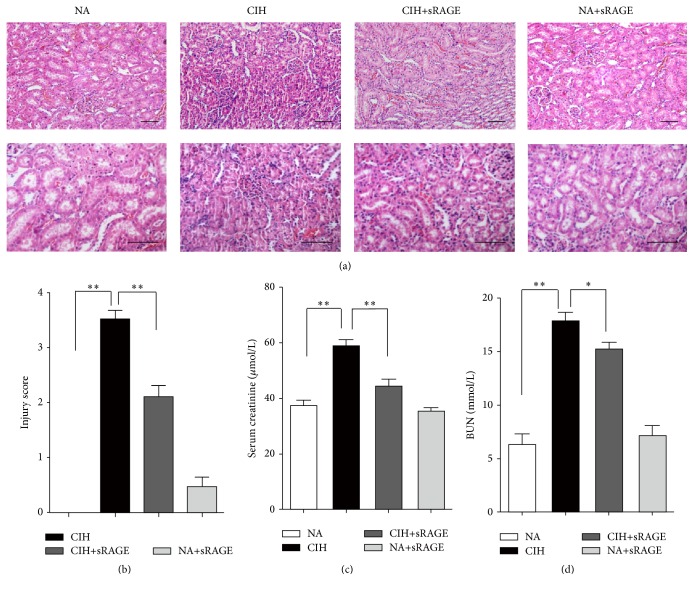
Effect of sRAGE on CIH-induced histological damage and renal dysfunction. (a) Representative kidney sections from normal air (NA) group, CIH group, CIH+sRAGE group, and negative control (NA+sRAGE) group are stained by H&E (scale bar: 50 *μ*m). In light microscopic examination, tubular degeneration, interstitial neutrophil infiltration, and massive desquamation of renal epithelium are more remarkable in the kidney tissues of CIH rats compared to NA group, while pretreatment of sRAGE apparently shows almost normal tubules and mild dilatation of tubular lumen absence of severe inflammatory infiltrations. (b) Sections are graded based on the 0–5 Jablonski grading scale averaging the values from 10 fields per kidney under microscopy. Renal function is determined by serum creatinine (c) and BUN (d). Data are presented as the mean ± SEM. ^*∗*^ *P* < 0.05, ^*∗∗*^ *P* < 0.01; *n* = 6/group.

In histological examination of kidney stained with hematoxylin-eosin, NA group rats showed normal glomerular and tubular structures, while CIH resulted in prominent tubular atrophy and inflammatory cell infiltration (Figure 1(a)). Injury score was further evaluated by the modified 0–5 Jablonski grading scale (Figure 1(b)). Consistent with desquamation of renal tubules epithelium in CIH group rats, the serum creatinine as well as BUN levels significantly elevated (58.59 ± 5.84 *μ*mol/L and 17.54 ± 1.97 mmol/L, *P* < 0.001) as compared to the NA control group (37.29 ± 5.07 *μ*mol/L and 6.12 ± 2.47 mmol/L; Figures 1(c) and 1(d)). However, animals treated with sRAGE before each hypoxia circle seldom displayed extensive features of tubule epithelial swelling and narrowed tubular lumens, without significant changes in distal convoluted tubule (Figure 1(a)). Contrast to CIH-incurred renal damage, sRAGE attenuated dysfunction and inflammation, as reflected by improvements in serum parameters (creatinine decreased by 24.41%, *P* = 0.0043; BUN decreased by 14.59%, *P* = 0.041) and histological grade (*P* = 0.002).

### 3.2. Soluble RAGE Attenuated CIH-Induced Renal Tubular Endothelial Cell Apoptosis

**Figure 2:**
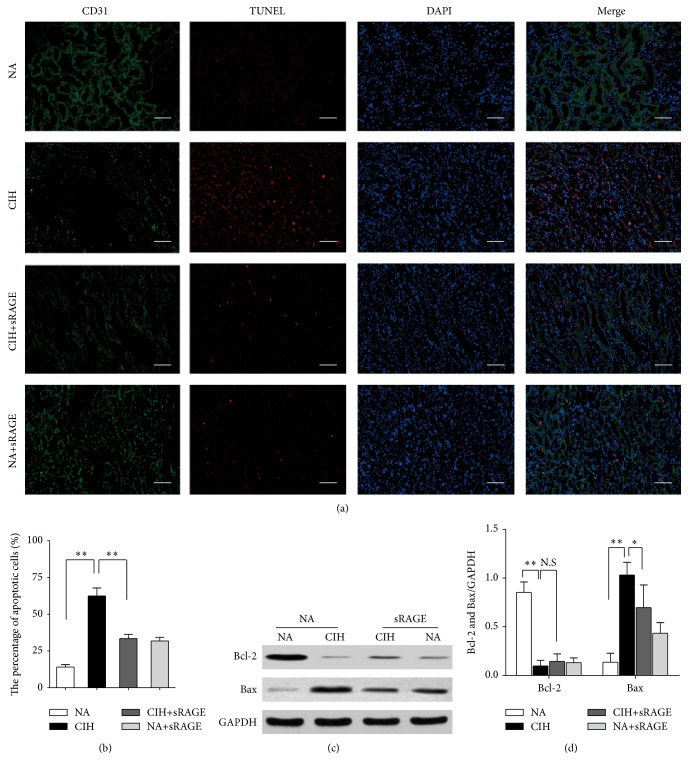
Effect of sRAGE on renal tubular endothelial cell apoptosis. (a) Representative immunofluorescence staining for CD31 (green), TUNEL (red), DAPI (blue), and the merged pictures from kidney tissues of each group. The scale bars represent 50 *μ*m. (b) Quantitative assessment of the percentage of apoptosis by counting the TUNEL^+^/DAPI^+^ cells in 10 random fields (100x) for each section. (c) Western blots analysis of Bcl-2 and Bax protein in comparison with GAPDH used as a loading control. (d) Representative bar diagram showing quantitative relative levels of Bcl-2 and Bax in NA, CIH, CIH+sRAGE, and NA plus sRAGE treated groups. Data are presented as the mean ± SEM. NS: no significance. ^*∗*^ *P* < 0.05, ^*∗∗*^ *P* < 0.01; *n* = 6/group.

Indeed, the renal tubular endothelial cell injury has been a key to the pathogenesis of CKD. Since CD31 was recognized to be a specific marker of endothelial cells [26], we performed immunofluorescence staining of CD31 and TUNEL to evaluate the degree of renal tubular endothelial cell apoptosis. DAPI was used to visualize cell nuclei; thus merged immunofluorescent TUNEL/DAPI staining depicted the proportion of apoptosis. In normal kidneys, CD31^+^ cells clearly stained in the wall of renal proximal and distal tubules did not express TUNEL^+^ cells. In contrast, CIH significantly reduced CD31 expression in corticomedullary junction and peritubular capillary endothelium, indicating that chronic hypoxia caused severe endothelial injury (Figure 2(a)). In addition, TUNEL^+^ cells were widely noted at the corticomedullary section in the CIH group. Colocalization of CD31/TUNEL immunofluorescent staining yielded that endothelial cells were undergoing apoptosis and the percentage of apoptotic endothelial cells was greatest following CIH exposure (Figure 2(a)). Upon pretreatment with sRAGE during CIH, only some faint, nonspecific, red background TUNEL staining was observed, whereas endothelial CD31 staining remained relatively apparent. The specific costaining for CD31 and lessened TUNEL^+^ cells in the merged picture suggested sRAGE ameliorated CIH-induced endothelial injury. In line with this, the percentage of apoptosis was significantly reduced 37% by sRAGE compared with the CIH group (*P* = 0.002, Figure 2(b)).

Oxidative stress affects the endothelial cell apoptosis by regulation of the balance between Bax and Bcl-2 proteins [27]. In contrast to NA group, expression of the proapoptotic protein Bax was upregulated during CIH process, whereas the magnitude of antiapoptotic protein Bcl-2 was significantly decreased as shown in Figure 2(c). Our histological results were further supported by Bcl-2/Bax protein ratio. Bcl-2/Bax ratio decreased from 1.49 ± 1.18 to 0.26 ± 0.43, suggestive of CIH-promoted propensity to apoptosis. But pretreatment of sRAGE exhibited a significant improvement of 125.11% in Bcl-2/Bax protein ratio compared with CIH alone. These data indicated that tubular endothelial cell apoptosis played a critical role in CIH-induced renal injury. Therefore, administration of sRAGE alleviated the renal endothelial cell death through a Bcl-2/Bax-dependent mechanism, thus improving functional recovery.

### 3.3. Effect of sRAGE on CIH-Induced HMGB1 Expression

**Figure 3:**
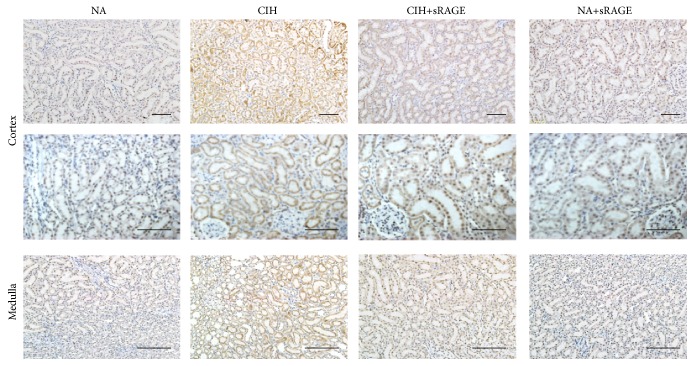
Representative immunohistochemistry of renal cortex (the top panel including glomeruli and proximal tubule) and medulla (the bottom panel including peritubular capillaries and distal tubule) for localization of HMGB1 (scale bar: 50 *μ*m). HMGB1 is abundant in cytoplasmic renal tubules of CIH group, compared with nuclear patterns of HMGB1 in NA group. sRAGE gradually attenuates HMGB1 cytoplasmic deposition, intraluminal infiltration, and nuclear staining in expanded renal tubules.

During oxidative stress provoked necrotic process, cells invariably lose membrane integrity and eventually lyse, resulting in intracellular contents release such as HMGB1. To ascertain it, immunohistochemistry was performed to determine the location of HMGB1 in each group. Results showed that HMGB1 expression was predominantly detected in cortical areas in contrast to medulla, meaning that proximal tubular cells were likely to be a prominent source of HMGB1. In CIH group, HMGB1 was expressed diffusely in the distended tubular cytoplasms as well as the nuclei of renal tubular epithelial cells, whereas HMGB1 was not or modestly expressed in the nuclei of proximal and distal convoluted tubules in NA group (Figure 3). In addition, the increased extracellular and cytoplasmic HMGB1 in CIH rats was gradually attenuated upon pretreatment of sRAGE, as depicted by lessened but mild expression in proximal and distal convoluted tubule (Figure 3). Negative control group (NA+sRAGE) excluded the possibility of nonspecific staining of sRAGE.

### 3.4. Effect of sRAGE on RAGE-HMGB1 Downstream Inflammatory Cytokines and Molecules

**Figure 4:**
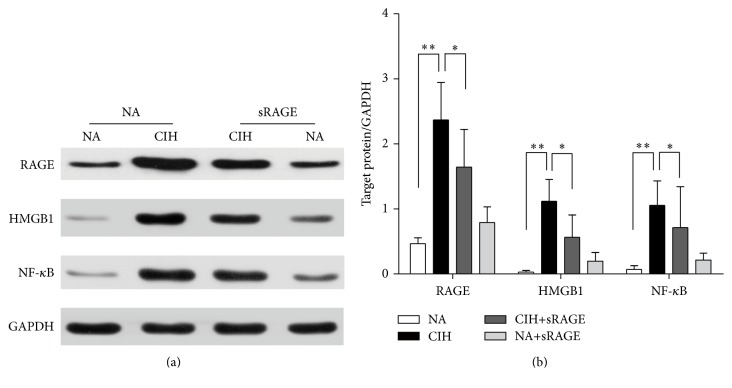
Effect of sRAGE on expressions of RAGE, HMGB1, and NF-*κ*B. (a) Representative western blot images (upper panel) and GAPDH (lower panel) used as the endogenous control are shown in each group. (b) Quantification densitometric analysis summarizes the fold changes of protein levels normalized to GAPDH. Data are expressed as means ± SEM. ^*∗*^ *P* < 0.05, ^*∗∗*^ *P* < 0.01; *n* = 6/group.

To distinguish the deleterious contribution of RAGE-HMGB1 in the pathogenesis of CIH-induced kidney injury, western blot technique was used to detect RAGE-HMGB1 and associated inflammatory molecules. Results demonstrated significant differences in RAGE-HMGB1 expression between NA and CIH control (RAGE: 0.466 ± 0.090 versus 2.368 ± 0.931, HMGB1: 0.038 ± 0.026 versus 1.118 ± 0.335, *P* < 0.001, Figure 4). Also, we found that NF-*κ*B was significantly increased in CIH group as compared to normal condition (0.071 ± 0.056 versus 1.056 ± 0.376, *P* < 0.001, Figure 4). In contrast to CIH alone, sRAGE-pretreatment group exhibited a significant suppression on RAGE (1.643 ± 0.581, *P* < 0.01, Figure 4) and also on HMGB1 (0.566 ± 0.341, *P* < 0.01, Figure 4). Besides, sRAGE played a pivotal role in the inhibition of NF-*κ*B activation (0.713 ± 0.628, *P* < 0.05, Figure 4). Conversely, treatment with sRAGE alone did not elicit apparent changes in RAGE-HMGB1 or NF-*κ*B activation. In this regard, engagement of RAGE-HMGB1 may accompany transcription factor NF-*κ*B activation that regulated the induction of multiple proinflammatory cytokines.

**Figure 5:**
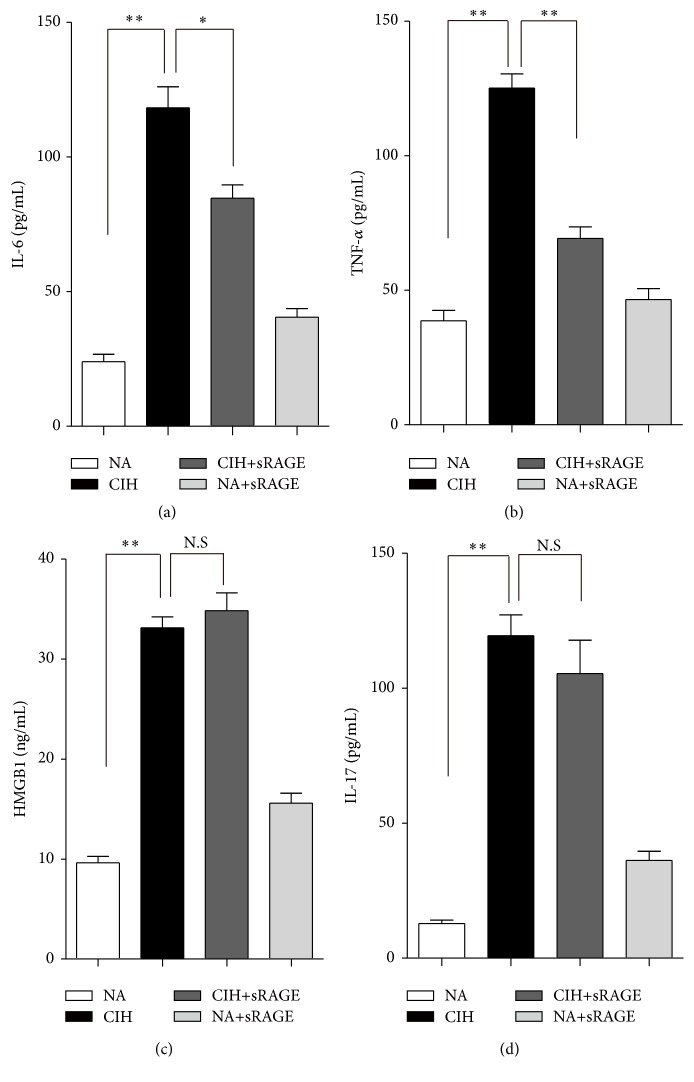
Effect of sRAGE on proinflammatory cytokines IL-6 (a), TNF-a (b), HMGB1 (c), and IL-17 (d) in serum of rats. Data are expressed as means ± SEM. NS: no significance. ^*∗*^ *P* < 0.05, ^*∗∗*^ *P* < 0.01; *n* = 6/group.

Following 5 weeks of CIH exposure, serum inflammatory cytokines of IL-6 and TNF-a increased significantly (118.28 ± 18.98 pg/mL and 125.16 ± 13.04 pg/mL resp., *P* < 0.0001; Figures 5(a) and 5(b)) but were lowly detectable in control (24.03 ± 6.77 pg/mL and 38.72 ± 9.36 pg/mL) and CIH+sRAGE group (84.75 ± 11.99 pg/mL and 69.29 ± 10.49 pg/mL). As expected, elevated serum levels of HMGB1 and IL-17 indeed occurred in the CIH rats (32.88 ± 2.69 ng/mL and 119.49 ± 18.77 pg/mL, *P* < 0.01; Figures 5(c) and 5(d)). Importantly, the results obtained from serum corresponded to the expression of RAGE signaling and inflammation response in tissues. Furthermore, as showed in Figures 5(a) and 5(b), the inhibitory effect of sRAGE on amplified inflammatory cytokine productions of IL-6 and TNF-*α* under CIH condition was obvious. However, it should be noteworthy that sRAGE had an adverse effect on the circulatory HMGB1 in CIH+sRAGE group rats compared with CIH alone (34.58 ± 4.32 ng/mL, *P* = 0.43; Figure 5(c)). Another negative result was observed in subsequent decreased levels of IL-17 in CIH+sRAGE group rats (105.49 ± 30.21 pg/mL, *P* = 0.061; Figure 5(d)). In this in vivo study, we confirmed the potential therapeutic effect of sRAGE on RAGE mediated inflammatory molecular signaling accompanied by reduced cytokines without the presence of circulatory HMGB1 and IL-17.

### 3.5. Soluble RAGE Modulates P-P38 and P-JNK but Not P-ERK Signaling

**Figure 6:**
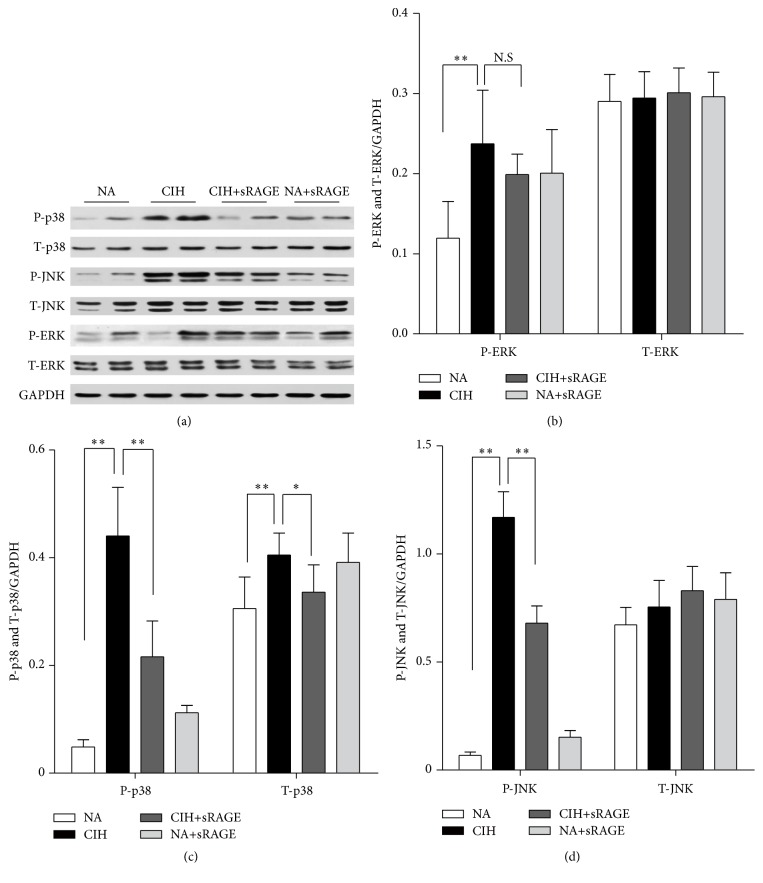
Representative western blot images show the effect of sRAGE on phosphorylated (P) and total (T) ERK, p38, and JNK expression (a). Histograms represent the quantitative densitometric ratio of MAPK signaling molecules ERK (b), p38 (c), and JNK (d) normalized to GAPDH in each group. Data are expressed as means ± SEM. NS: no significance. ^*∗*^ *P* < 0.05, ^*∗∗*^ *P* < 0.01; *n* = 6/group.

Mitogen-activated protein kinases (MAPKs) are well accepted upstream modulators of apoptosis and inflammatory cytokines. They also have crucial roles in signal transduction from the cell surface to the nucleus, which are required for subsequent NF-*κ*B transcriptional activation. As shown in Figure 6(a), the phosphorylated JNK and p38, measured as phospho/total-JNK and phospho/total-p38 level, both reached maximal kinase activities after 5 weeks of CIH exposure (JNK: 1.75 ± 0.81 and p38: 1.11 ± 0.49, *P* < 0.01). Also, CIH tended to enhance the phosphorylation of ERK1/2 approximately twofold over basal levels (0.24 ± 0.16 versus 0.12 ± 0.11, *P* = 0.013, Figure 6(b)). Regarding these, MAPKs family including p38, JNK, and ERK1/2 were investigated to be activated in response to oxidative stress. To address whether sRAGE could modulate MAPK activity, we further used specific antibodies to establish the active forms of the kinases activities. In contrast to CIH alone, the levels of phosphorylated JNK and p38 along with sRAGE treatment were decreased (JNK: 0.87 ± 0.31 and p38: 0.69 ± 0.61, *P* < 0.01; Figures 6(c) and 6(d)), whereas the phosphorylation levels of p-ERK1/2 were not significantly affected (0.25 ± 0.09, *P* > 0.05, Figure 6(b)). sRAGE treatment abrogated t-p38 activation, but no changes in the total levels of ERK1/2 or JNK were detectable.

Accordingly, we identify RAGE ligand as key mediators of MAPK downstream molecules leading to CIH-activated inflammation and apoptosis, while signaling proteins such as p38 and JNK MAP kinases are potentially regulators involved in the renal protection of sRAGE.

## 4. Discussion

Accumulating evidences indicated that RAGE contributed, at least in part, to the development of OSA complications, such as diabetes and nephropathy, cardiovascular disease, and chronic inflammation [28]. Recent studies provided insight into sRAGE in competing with cell surface RAGE for ligand binding, thus potentially representing a novel molecular target for OSA-associated chronic kidney disease. The present study shows that RAGE-HMGB1 plays a pivotal role in a CIH model. Furthermore, it is the first evidence that sRAGE demonstrates its anti-inflammatory and antiapoptotic effects by altering p38 and JNK signaling pathways.

Histological examination confirmed that our CIH protocol was sufficient to trigger renal damage. It has been shown that HIF1-*α* is the main molecular effector of hypoxia signaling and able to combine the HIF-1*α* binding site present in the RAGE promoter region [29]. Thereby hypoxia may activate RAGE mRNA gene transcription and stimulate RAGE production. Renal interstitial and tubular endothelial cells express specific RAGEs, ongoing generation of which may amplify chronic cellular perturbation and oxidant stress damage by engagement of these receptors on the endothelial surfaces [30]. Our observations in colocalization of CD31/TUNEL immunofluorescent staining revealed that activation of RAGE accelerated tubular endothelial apoptosis. Apart from a possible adaptive response to chronic hypoxia, activation of RAGE observed in our CIH model probably contributed to the CIH-induced injury according to western blot analysis. Of special interest is the finding of extracellular and abundant cytoplasmic accumulations of HMGB1 following CIH. Our results demonstrated that HMGB1 was limited in the nucleus of renal parenchyma cells under normal condition, but dramatically translocated into the cytoplasm and extracellular matrix upon hypoxia insult. For one reason, HMGB1 is passively released in response to inflammatory stress or necrosis [31]. For another, translocation of HMGB1 from the nucleus to cytoplasm requires inflammasome and caspase activity, thus facilitating the chronic inflammation and apoptosis [32, 33]. Furthermore, HMGB1 can behave as a secreted cytokine promoting neutrophil accumulating [34] and activate macrophages/monocytes to release more proinflammatory cytokines [35, 36]. Consistently, both ELISA and immunohistochemistry results showed that HMGB1 secreted from serum and tissue was collectively elevated and correlated with upregulated TNF-*α* and IL-6 after CIH. In addition, as a widely acknowledged cytokine for regulating inflammatory reaction and leukocyte migration, IL-17 was reported to be upregulated in RAGE-HMGB1 associated injury [37]. Report from Akirav et al. also indicated the association of RAGE expression and increased IL-17 [38]. Moreover, HMGB1 contributed to lymphocyte infiltration and the release of the Th17 cell specific cytokine IL-17 [39]. In our research, we confirmed previous results, exactly supporting the positive feedback loops of RAGE-HMGB1 activation and proinflammatory mediators.

RAGE-mediated cascades of signal transduction could promote the proinflammatory NF-*κ*B and the MAPK pathway in endothelial cells and monocytes. Importantly, the pathogenic role of RAGE appears to depend on the level of NF-*κ*B transcriptional activity [40]. Inhibition of NF-*κ*B decreased cardiomyocyte apoptosis and recruitment of neutrophils accompanied with HMGB1 suppression [41], indicative of the reciprocal modulation of NF-*κ*B and HMGB1. A range of animal models in vitro and in vivo have demonstrated the involvement of RAGE in pathophysiologic processes, using a receptor decoy such as sRAGE [42]. Phosphorylated levels of p38, JNK, and ERK in present study were higher after CIH exposure and subsequently affected by sRAGE to different extent, implicating RAGE ligand as key mediators in MAPKs signaling. In line with our results, there was evidence in rat renal tubular epithelial cells that indicated the critical importance of HMGB1 in inducing circulating cyto/chemokines secretion through MAP kinase pathways [43]. Similar results were also observed in early reports that RAGE induced NF-*κ*B activation and IL-1 and TNF-*α* production were dependent on p38 phosphorylation in diabetic glomerular injury [44, 45]. In addition, RAGE ligand interaction may directly induce generation of ERK and reactive oxygen species [46]. Specially, in our studies, these responses of MAPKs to sRAGE lack the participation of p-ERK1/2. Consistent with our results, Taguchi et al. found blockage of RAGE-amphoterin interaction also suppressed p38 and SAP/JNK MAPKs [47]. On the contrary, inhibition of RAGE by siRNA could reduce phosphorylated-ERK in cyst formation [48]. This ambiguity regarding MAPK molecular mechanisms perhaps depends on the cell and RAGE ligand types in vivo and in vitro.

sRAGE can be used as a biomarker in RAGE-dependent inflammations as well as a therapeutic agent to neutralize hypoxia induced inflammation [49]. Moreover, sRAGE can cancel the effects of AGEs on cells in culture [50]. In another hypoxia/reoxygenation model, sRAGE significantly decreased cellular lactic dehydrogenase leakage and increased cell viability in neonatal rat cardiomyocytes [51]. The published data suggest that application of sRAGE is identified to intercept RAGE ligand interaction and subsequent downstream signaling [52]. Since sustained MAPK activation has been associated with oxidative stress and cell apoptosis [53], through histologic and western blot analysis, we reasoned that sRAGE protected against renal inflammation and apoptosis by suppression of p38 and JNK MAPK signaling molecules.

Previous studies revealed the decreased sRAGE levels increased the propensity toward chronic inflammation such as hypertension [54] and coronary artery disease [55]. Serum sRAGE levels were elevated significantly in patients with decreased renal function and inversely related to inflammation [21]. These observations lead us to propose that subsequent production of sRAGE potentially protects against the decreased renal function, but Kalousová et al. found it was not related to mortality of haemodialysis patients [56]. Whether sRAGE represents only an epiphenomenon or a compensatory protective mechanism is still unknown. Although the protective effect of sRAGE is not as effective as the RAGE-deletion [57], the property of long half-life after intraperitoneal injection into normal rats renders the sustained effect in each hypoxia cycle until the end of the observation [58]. Considering RAGE is a multiligand receptor, the accurate blocking target of sRAGE remains to be elucidated. For example, sRAGE is found to interact with Mac-1 in an HMGB1-induced arthritis model [59]. In terms of proinflammatory and proapoptotic effects, HMGB1 is likely to be a main target of sRAGE [60]. Since S100 proteins and HMGB1 certainly do not exclusively bind to RAGE [61], sRAGE did not only result from intercepting the interaction of ligands with cell surface RAGE, but with other possible receptors. That was why that we only observed upregulated circulatory HMGB1 in serum without suddenly degraded levels by sRAGE. It is reasonably speculated that HMGB1 passively released from nucleus to circulation might not be efficiently scavenged. However, in accordance with immunohistochemistry results, western blot analysis of total renal cellular lysates detected the difference of HMGB1 between CIH and exogenous administration of sRAGE groups, suggesting that sRAGE exerted its effect by downregulation of HMGB1. Remarkably, Lee et al. determined that sRAGE exhibited no toxic effects on the liver by testing the activity of ALT [10], providing additional support for this potential therapeutic strategy.

A limitation of this study was the lack of verification that whether the decreased RAGE expression induced by sRAGE treatment was abrogated with exogenous HMBG1 administration. We did not observe such endogenous sRAGE level in renal insufficiency following the chronic hypoxia. To confirm its exact blockage target of RAGE ligand interaction, the capacity of sRAGE in inflammatory responses from diverse models remains to be elucidated.

## 5. Conclusions

Taken together, RAGE and its ligand HMGB1 activate chronic inflammatory transduction cascades that contribute to the pathogenesis of CIH-induced renal injury. The consequences of amplifying inflammatory response include the recruitment of inflammatory cytokines and effector molecules (sustained expression of NF-*κ*B, TNF-*α*, IL-6, and MAPK signaling), leading to apoptosis and accelerated renal dysfunction. Interruption of RAGE interaction by administration of sRAGE has been shown to attenuate these detrimental effects. According to a decoy mechanism, blockade of RAGE ligand interaction could provide a new therapeutic approach in the development and progression of OSA-associated chronic kidney disease.

## References

[1] Nicholl D. D. M., Ahmed S. B., Loewen A. H. S. (2012). Clinical presentation of obstructive sleep apnea in patients with chronic kidney disease. *Journal of Clinical Sleep Medicine*.

[2] Kimmel P. L., Miller G., Mendelson W. B. (1989). Sleep apnea syndrome in chronic renal disease. *The American Journal of Medicine*.

[3] Sim J. J., Rasgon S. A., Kujubu D. A. (2009). Sleep apnea in early and advanced chronic kidney disease: Kaiser Permanente Southern California cohor. *Chest*.

[4] Hanly P. J., Ahmed S. B. (2014). Sleep apnea and the kidney: is sleep apnea a risk factor for chronic kidney disease?. *Chest*.

[5] Beecroft J. M., Pierratos A., Hanly P. J. (2009). Clinical presentation of obstructive sleep apnea in patients with end-stage renal disease. *Journal of Clinical Sleep Medicine*.

[6] Chiang A. A. (2006). Obstructive sleep apnea and chronic intermittent hypoxia: a review. *Chinese Journal of Physiology*.

[7] Stern D., Yan S. D., Yan S. F., Schmidt A. M. (2002). Receptor for advanced glycation endproducts: a multiligand receptor magnifying cell stress in diverse pathologic settings. *Advanced Drug Delivery Reviews*.

[8] Wu K.-M., Lin C.-C., Chiu C.-H., Liaw S.-F. (2010). Effect of treatment by nasal continuous positive airway pressure on serum high mobility group box-1 protein in obstructive sleep apnea. *Chest*.

[9] Duru S., Hikmet Firat I., Colak N., Giniş Z., Delibaşi T., Ardiç S. (2012). Serum S100B protein: a useful marker in obstructive sleep apnea syndrome. *Neurologia i Neurochirurgia Polska*.

[10] Lee E. J., Park E. Y., Mun H. (2015). Soluble receptor for advanced glycation end products inhibits disease progression in autosomal dominant polycystic kidney disease by down-regulating cell proliferation. *The FASEB Journal*.

[11] D'Agati V., Schmidt A. M. (2010). RAGE and the pathogenesis of chronic kidney disease. *Nature Reviews Nephrology*.

[12] Bao Y.-S., Na S.-P., Zhang P. (2012). Characterization of interleukin-33 and soluble ST2 in serum and their association with disease severity in patients with chronic kidney disease. *Journal of Clinical Immunology*.

[13] Zhu P., Xie L., Ding H.-S., Gong Q., Yang J., Yang L. (2013). High mobility group box 1 and kidney diseases (Review). *International Journal of Molecular Medicine*.

[14] Schmidt A. M., Yan S. D., Yan S. F., Stern D. M. (2001). The multiligand receptor RAGE as a progression factor amplifying immune and inflammatory responses. *The Journal of Clinical Investigation*.

[15] Bruchfeld A., Qureshi A. R., Lindholm B. (2008). High mobility group box protein-1 correlates with renal function in chronic kidney disease (CKD). *Molecular Medicine*.

[16] Zhou T.-B. (2014). Role of high mobility group box 1 and its signaling pathways in renal diseases. *Journal of Receptors and Signal Transduction*.

[17] Leonardis D., Basta G., Mallamaci F. (2012). Circulating soluble receptor for advanced glycation end product (sRAGE) and left ventricular hypertrophy in patients with chronic kidney disease (CKD). *Nutrition, Metabolism and Cardiovascular Diseases*.

[18] Park E. Y., Kim B. H., Lee E. J. (2014). Targeting of receptor for advanced glycation end products suppresses cyst growth in polycystic kidney disease. *The Journal of Biological Chemistry*.

[19] Li J., Gong Q., Zhong S. (2011). Neutralization of the extracellular HMGB1 released by ischaemic damaged renal cells protects against renal ischaemia-reperfusion injury. *Nephrology Dialysis Transplantation*.

[20] Vazzana N., Santilli F., Cuccurullo C., Davì G. (2009). Soluble forms of RAGE in internal medicine. *Internal and Emergency Medicine*.

[21] Kalousová M., Hodková M., Kazderová M. (2006). Soluble receptor for advanced glycation end products in patients with decreased renal function. *American Journal of Kidney Diseases*.

[22] Angelo M. F., Aguirre A., Reyes R. X. A. (2014). The proinflammatory RAGE/NF-*κ*B pathway is involved in neuronal damage and reactive gliosis in a model of sleep apnea by intermittent hypoxia. *PLoS ONE*.

[23] Fu C., Jiang L., Zhu F. (2015). Chronic intermittent hypoxia leads to insulin resistance and impaired glucose tolerance through dysregulation of adipokines in non-obese rats. *Sleep and Breathing*.

[24] Farmer D. G. S., Ewart M.-A., Mair K. M., Kennedy S. (2014). Soluble receptor for advanced glycation end products (sRAGE) attenuates haemodynamic changes to chronic hypoxia in the mouse. *Pulmonary Pharmacology and Therapeutics*.

[25] Islam C. F., Mathie R. T., Dinneen M. D., Kiely E. A., Peters A. M., Grace P. A. (1997). Ischaemia-reperfusion injury in the rat kidney: the effect of preconditioning. *British Journal of Urology*.

[26] Nakao A., Neto J. S., Kanno S. (2005). Protection against ischemia/reperfusion injury in cardiac and renal transplantation with carbon monoxide, biliverdin and both. *American Journal of Transplantation*.

[27] Bagci E. Z., Vodovotz Y., Billiar T. R., Ermentrout G. B., Bahar I. (2006). Bistability in apoptosis: roles of Bax, Bcl-2, and mitochondrial permeability transition pores. *Biophysical Journal*.

[28] Bierhaus A., Humpert P. M., Morcos M. (2005). Understanding RAGE, the receptor for advanced glycation end products. *Journal of Molecular Medicine*.

[29] Pichiule P., Chavez J. C., Schmidt A. M., Vannucci S. J. (2007). Hypoxia-inducible factor-1 mediates neuronal expression of the receptor for advanced glycation end products following hypoxia/ischemia. *The Journal of Biological Chemistry*.

[30] Basta G., Lazzerini G., Massaro M. (2002). Advanced glycation end products activate endothelium through signal-transduction receptor RAGE: a mechanism for amplification of inflammatory responses. *Circulation*.

[31] Faraco G., Fossati S., Bianchi M. E. (2007). High mobility group box 1 protein is released by neural cells upon different stresses and worsens ischemic neurodegeneration *in vitro* and *in vivo*. *Journal of Neurochemistry*.

[32] Gwak G.-Y., Moon T. G., Lee D. H., Yoo B. C. (2012). Glycyrrhizin attenuates HMGB1-induced hepatocyte apoptosis by inhibiting the p38-dependent mitochondrial pathway. *World Journal of Gastroenterology*.

[33] Kim J.-B., Joon S. C., Yu Y.-M. (2006). HMGB1, a novel cytokine-like mediator linking acute neuronal death and delayed neuroinflammation in the postischemic brain. *The Journal of Neuroscience*.

[34] Oyama Y., Hashiguchi T., Taniguchi N. (2010). High-mobility group box-1 protein promotes granulomatous nephritis in adenine-induced nephropathy. *Laboratory Investigation*.

[35] Andersson U., Wang H., Palmblad K. (2000). High mobility group 1 protein (HMG-1) stimulates proinflammatory cytokine synthesis in human monocytes. *The Journal of Experimental Medicine*.

[36] Lau A., Wang S., Liu W., Haig A., Zhang Z.-X., Jevnikar A. M. (2014). Glycyrrhizic acid ameliorates HMGB1-mediated cell death and inflammation after renal ischemia reperfusion injury. *American Journal of Nephrology*.

[37] Jhun J. Y., Lee S. H., Kim H. Y. (2015). HMGB1/RAGE induces IL-17 expression to exaggerate inflammation in peripheral blood cells of hepatitis B patients. *Journal of Translational Medicine*.

[38] Akirav E. M., Preston-Hurlburt P., Garyu J. (2012). RAGE expression in human T cells: a link between environmental factors and adaptive immune responses. *PLoS ONE*.

[39] Lee C.-C., Lai Y.-T., Chang H.-T. (2013). Inhibition of high-mobility group box 1 in lung reduced airway inflammation and remodeling in a mouse model of chronic asthma. *Biochemical Pharmacology*.

[40] Villarreal A., Aviles Reyes R. X., Angelo M. F., Reinesf A. G., Ramos A. J. (2011). S100B alters neuronal survival and dendrite extension via RAGE-mediated NF-*κ*B signaling. *Journal of Neurochemistry*.

[41] Shi Z., Lian A., Zhang F. (2014). Nuclear factor-*κ*B activation inhibitor attenuates ischemia reperfusion injury and inhibits Hmgb1 expression. *Inflammation Research*.

[42] Wendt T. M., Tanji N., Guo J. (2003). RAGE drives the development of glomerulosclerosis and implicates podocyte activation in the pathogenesis of diabetic nephropathy. *American Journal of Pathology*.

[43] Simm A., Münch G., Seif F. (1997). Advanced glycation endproducts stimulate the MAP-kinase pathway in tubulus cell line LLC-PK1. *FEBS Letters*.

[44] Adhikary L., Chow F., Nikolic-Paterson D. J. (2004). Abnormal p38 mitogen-activated protein kinase signalling in human and experimental diabetic nephropathy. *Diabetologia*.

[45] Wu C.-H., Huang C.-M., Lin C.-H., Ho Y.-S., Chen C.-M., Lee H.-M. (2002). Advanced glycosylation end products induce NF-*κ*B dependent iNOS expression in RAW 264.7 cells. *Molecular and Cellular Endocrinology*.

[46] Lander H. M., Tauras J. M., Ogiste J. S., Hori O., Moss R. A., Schmidt A. M. (1997). Activation of the receptor for advanced glycation end products triggers a p21(ras)-dependent mitogen-activated protein kinase pathway regulated by oxidant stress. *The Journal of Biological Chemistry*.

[47] Taguchi A., Blood D. C., del Toro G. (2000). Blockade of RAGE-amphoterin signalling suppresses tumour growth and metastases. *Nature*.

[48] Park E. Y., Seo M. J., Park J. H. (2010). Effects of specific genes activating RAGE on polycystic kidney disease. *American Journal of Nephrology*.

[49] Iannitti R. G., Casagrande A., De Luca A. (2013). Hypoxia promotes danger-mediated inflammation via receptor for advanced glycation end products in cystic fibrosis. *American Journal of Respiratory and Critical Care Medicine*.

[50] Bowman M. A. H., Schmidt A. M. (2013). The next generation of RAGE modulators: implications for soluble RAGE therapies in vascular inflammation. *Journal of Molecular Medicine*.

[51] Guo C., Zeng X., Song J. (2012). A soluble receptor for advanced glycation end-products inhibits hypoxia/reoxygenation-induced apoptosis in rat cardiomyocytes via the mitochondrial pathway. *International Journal of Molecular Sciences*.

[52] Raucci A., Cugusi S., Antonelli A. (2008). A soluble form of the receptor for advanced glycation endproducts (RAGE) is produced by proteolytic cleavage of the membrane-bound form by the sheddase a disintegrin and metalloprotease 10 (ADAM10). *The FASEB Journal*.

[53] Gong G., Xiang L., Yuan L. (2014). Protective effect of glycyrrhizin, a direct HMGB1 inhibitor, on focal cerebral ischemia/reperfusion-induced inflammation, oxidative stress, and apoptosis in rats. *PLoS ONE*.

[54] Geroldi D., Falcone C., Emanuele E. (2005). Decreased plasma levels of soluble receptor for advanced glycation end-products in patients with essential hypertension. *Journal of Hypertension*.

[55] Chiang K.-H., Huang P.-H., Huang S.-S., Wu T.-C., Chen J.-W., Lin S.-J. (2009). Plasma levels of soluble receptor for advanced glycation end products are associated with endothelial function and predict cardiovascular events in nondiabetic patients. *Coronary Artery Disease*.

[56] Kalousová M., Jáchymová M., Oto M. (2007). Receptor for advanced glycation end products—soluble form and gene polymorphisms in chronic haemodialysis patients. *Nephrology Dialysis Transplantation*.

[57] Liliensiek B., Weigand M. A., Bierhaus A. (2004). Receptor for advanced glycation end products (RAGE) regulates sepsis but not the adaptive immune response. *The Journal of Clinical Investigation*.

[58] Renard C., Chappey O., Wautier M.-P. (1997). Recombinant advanced glycation end product receptor pharmacokinetics in normal and diabetic rats. *Molecular Pharmacology*.

[59] Daffu G., del Pozo C. H., O'Shea K. M., Ananthakrishnan R., Ramasamy R., Schmidt A. M. (2013). Radical roles for RAGE in the pathogenesis of oxidative stress in cardiovascular diseases and beyond. *International Journal of Molecular Sciences*.

[60] Jiang W., Bell C. W., Pisetsky D. S. (2007). The relationship between apoptosis and high-mobility group protein 1 release from murine macrophages stimulated with lipopolysaccharide or polyinosinic-polycytidylic acid. *The Journal of Immunology*.

[61] Robinson M. J., Tessier P., Poulsom R., Hogg N. (2002). The S100 family heterodimer, MRP-8/14, binds with high affinity to heparin and heparan sulfate glycosaminoglycans on endothelial cells. *The Journal of Biological Chemistry*.

